# Supplementary material for: Mercury isotope evidence for Arctic summertime re-emission of mercury from the cryosphere
Source: Nat Commun. 2022 Aug 24;13:4956. doi: 10.1038/s41467-022-32440-8 (PMC9402541; doi:10.1038/s41467-022-32440-8)
Supplement: Supplementary file 1 — Supplementary Information [file 41467_2022_32440_MOESM1_ESM.pdf]

Supplementary Information for

Mercury isotope evidence for Arctic summertime re-emission of mercury from the cryosphere

Beatriz Ferreira Araujo[#1], Stefan Osterwalder[2,3#*], Natalie Szponar[4#], Domenica Lee[4], Mariia V. Petrova[5], Jakob Boyd Pernov[6,7], Shaddy Ahmed[3], Lars-Eric Heimbürger-Boavida[5], Laure Laffont[1], Roman Teisserenc[8], Nikita Tananaev[9,10], Claus Nordstrom[6], Olivier Magand[3], Geoff Stupple[11], Henrik Skov[6], Alexandra Steffen[11], Bridget Bergquist[4], Katrine Aspmo Pfaffhuber[12], Jennie L. Thomas[3], Simon Scheper[13,14], Tuukka Petäjä[15], Aurélien Dommergue[3], Jeroen E. Sonke[1*]

[1] Géosciences Environnement Toulouse, CNRS, IRD, Université de Toulouse, France
[2] Institute of Agricultural Sciences, ETH Zurich, Zurich, Switzerland
[3] Univ. Grenoble Alpes, CNRS, IRD, Grenoble INP, IGE, Grenoble, France
[4] Department of Earth Sciences, University of Toronto, Ontario, Canada
[5] CNRS/INSU, Aix Marseille Université, Université de Toulon, IRD, Mediterranean Institute of Oceanography, Marseille, France
[6] Department of Environmental Science, iClimate, Aarhus University, Roskilde, Denmark.
[7] Extreme Environments Research Laboratory, École Polytechnique fédérale de Lausanne, Sion, Switzerland
[8] Laboratoire Écologie Fonctionnelle et Environnement, UMR5245 CNRS/UPS/INPT, Toulouse, France
[9] Melnikov Permafrost Institute, Siberian Branch, Russian Academy of Sciences, Yakutsk, Russia
[10] Institute of Natural Sciences, North-Eastern Federal University, Yakutsk, Russia
[11] Environment and Climate Change Canada, Air Quality Research Division, Toronto, Canada
[12] Norwegian Institute for Air Research, Kjeller, Norway.
[13] Dr. Simon Scheper - Research | Consulting | Teaching, Dähre, Germany
[14] Environmental Geosciences, University of Basel, Basel, Switzerland
[15] Institute for Atmospheric and Earth System Research, University of Helsinki, Helsinki, Finland

[#] these authors contributed equally as 1st authors: Beatriz Ferreira Araujo, Stefan Osterwalder, Natalie Szponar
* corresponding authors: stefan.osterwalder@usys.ethz.ch; jeroen.sonke@get.omp.eu

Contents:

Supplementary Note p.2

Supplementary Figures p.3

Supplementary Tables p.9

Supplementary References p.11

Supplementary Note

Literature data reported in Figure 4: Hg^0 [1–8], river Hg [9], snow Hg [7,8,10,11], lichens/moss [12].

Supplementary Figures

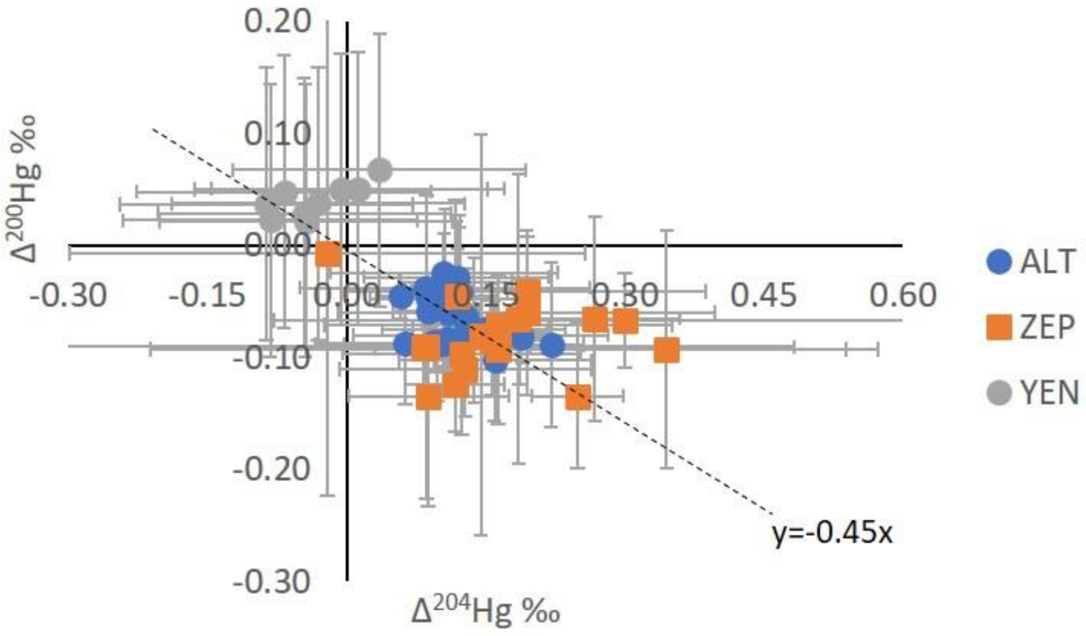

Supplementary Figure 1 Relationship between even Hg isotope MIF signatures Δ^{204}Hg and Δ^{200}Hg. The displayed data represent atmospheric Hg^0 at Zeppelin (ZEP) and at Alert (ALT) and dissolved Hg in the Yenisei River (YEN). Error bars represent the 2 SD uncertainties of individual samples.

a b

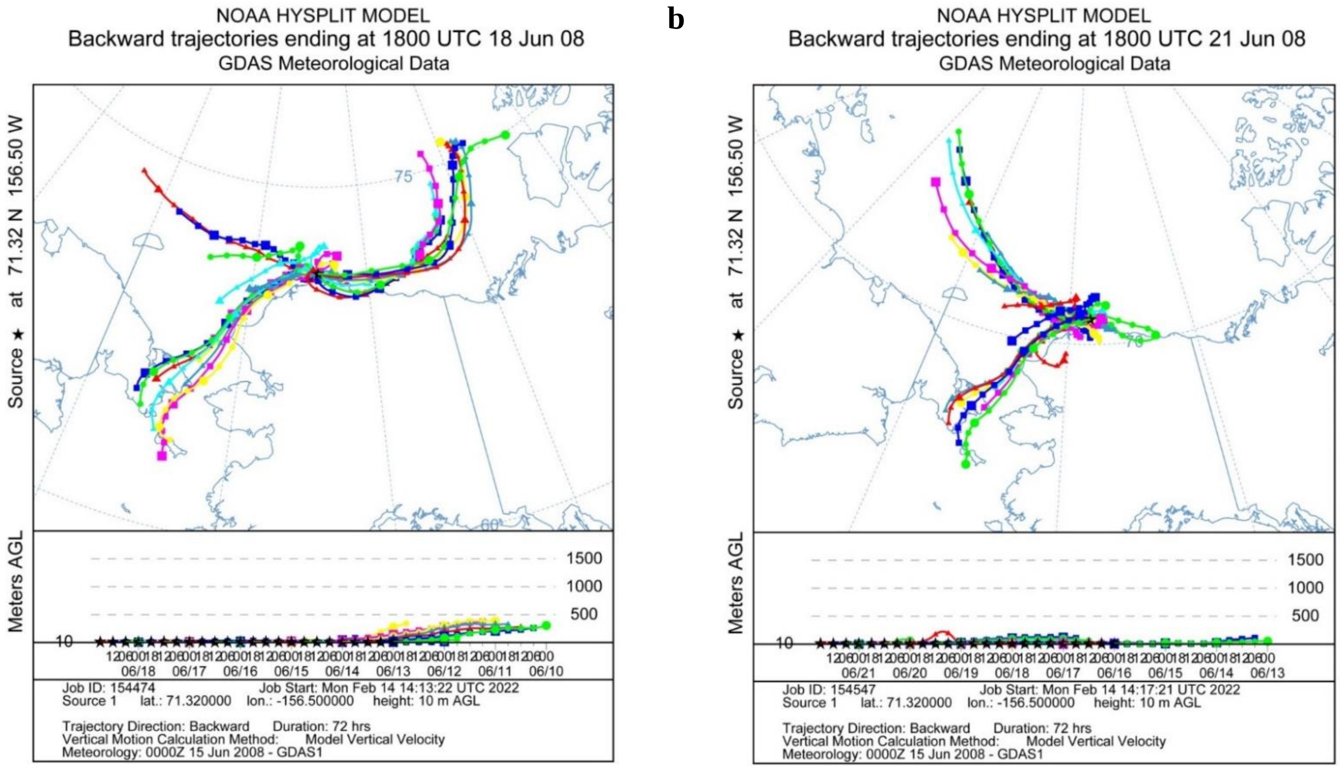

Supplementary Figure 2 Backward trajectory created using the NOAA HYSPLIT model. The 3h-back trajectories are drawn for the **(a)** June 15-18 2008 and **(b)** June 18-21 2008 Hg^0 isotope observation by [7] at Utqiagvik, USA. Maps were created by the authors using the HYSPLIT model https://www.arl.noaa.gov/hysplit/) and ready website (https://www.ready.noaa.gov) with permission from the NOAA Air Resources Laboratory's.

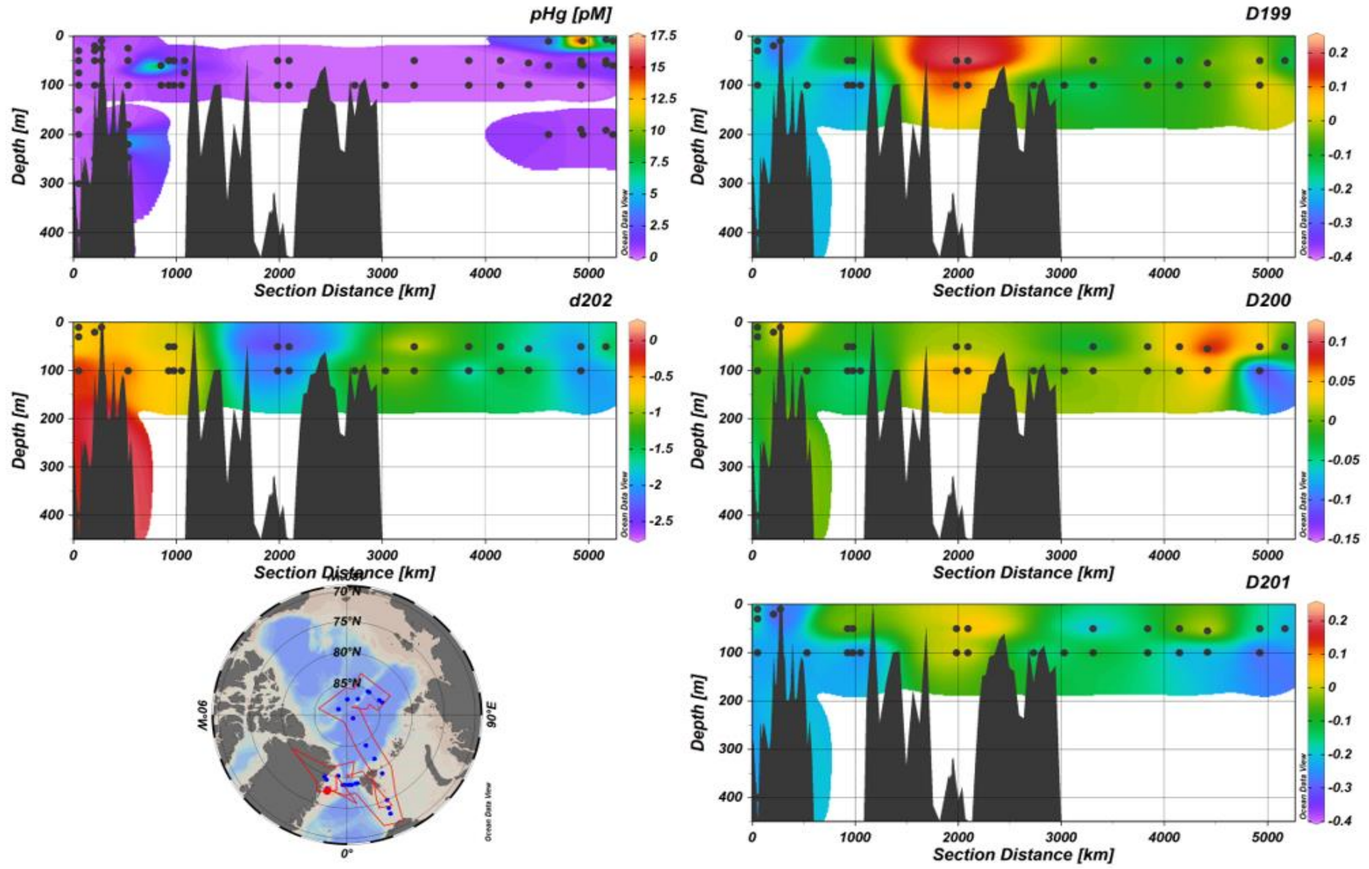

Supplementary Figure 3 Surface Arctic Ocean particulate Hg (pHg) concentrations, δ^{202}Hg, Δ^{199}Hg, Δ^{200}Hg, and Δ^{201}Hg signatures (in ‰). The data were obtained along a stretched cruise transect from the West Greenland coast to Svalbard, into the Barents Sea, and up to the central Arctic Ocean. Fram Strait data partially from [13]. The figure was created using Ocean Data View [14] with permission from Alfred Wegener Institute, Helmholtz Centre for Polar and Marine Research (AWI) (https://odv.awi.de/).

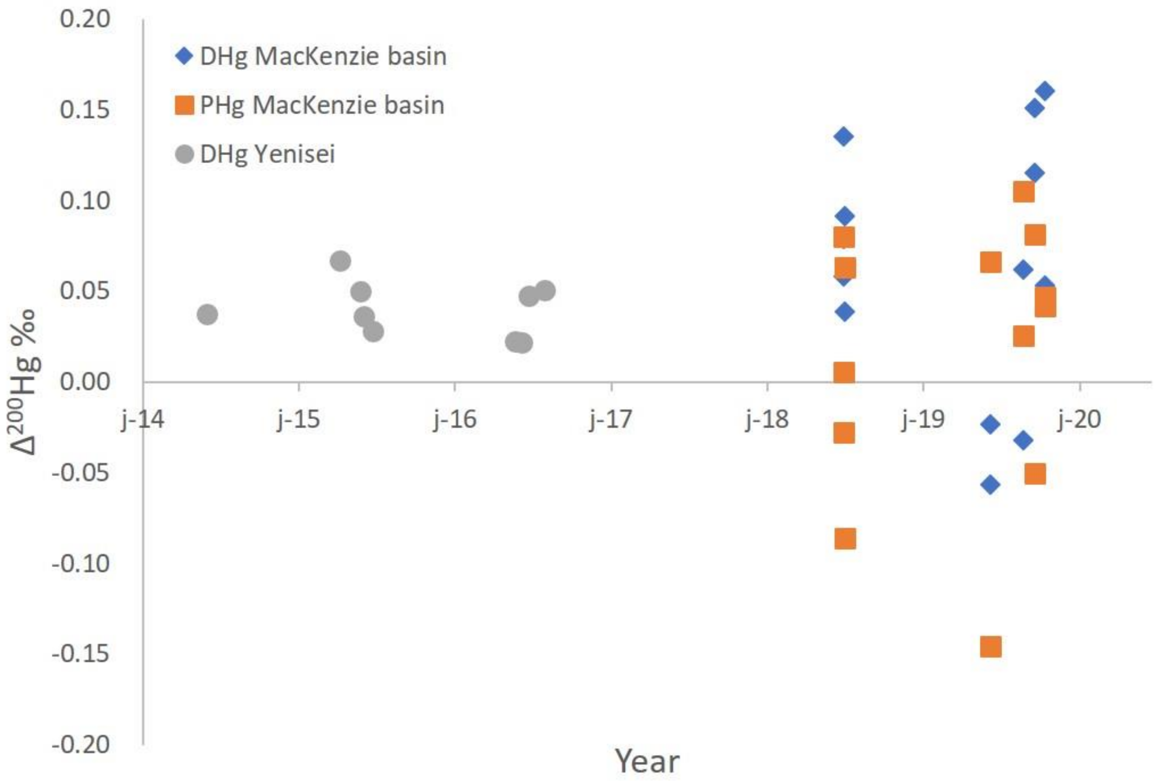

Supplementary Figure 4 Hg isotope observations on Arctic river dissolved (dHg) and particulate Hg (pHg). Mackenzie River (data near mouth, >65.5^{o} N, [9]) and Yenisei River (this study) samples were collected between 2014 and 2019.

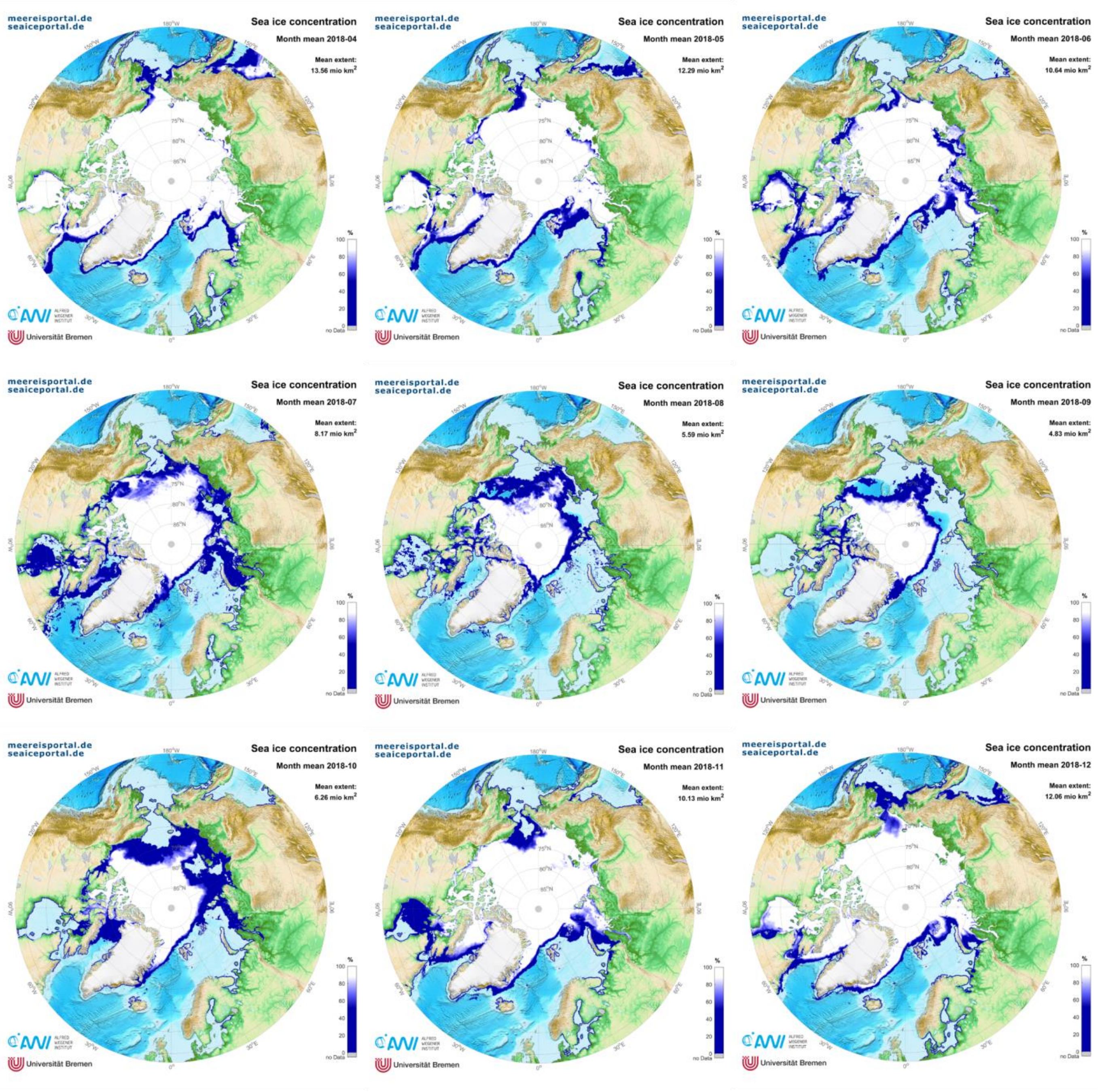

Supplementary Figure 5 Monthly mean sea ice concentration (%) from April 2018 through December 2018. Sea ice concentration data from April 01 to December 31, 2018 were obtained from https://www.meereisportal.de (grant: REKLIM-2013-04). The online sea-ice knowledge and data platform meereisportal.de is used with permission from Alfred Wegener Institute for Polar and Marine Research & German Society of Polar Research.

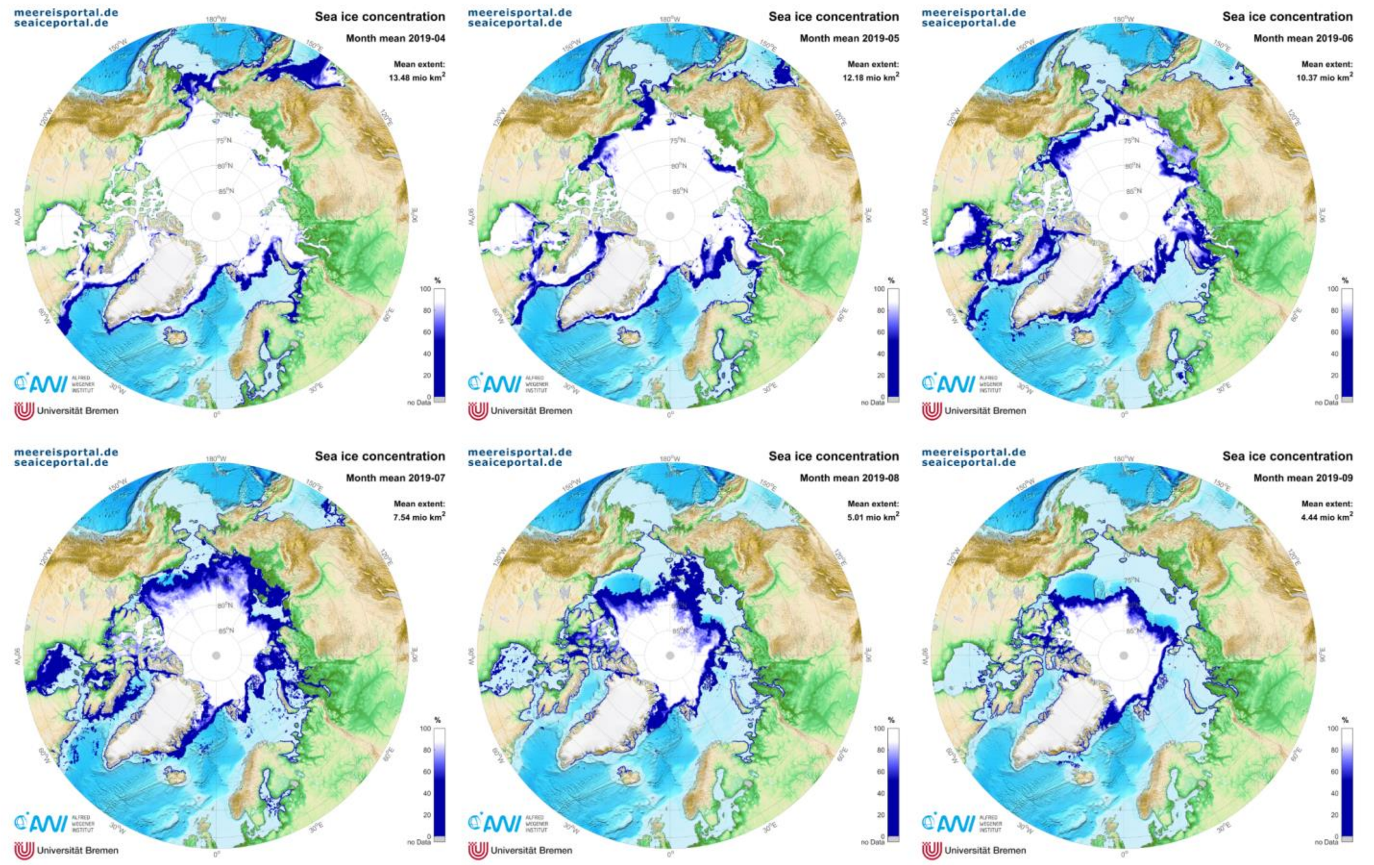

Supplementary Figure 6 Monthly mean sea ice concentration (%) from April 2019 through September 2019. Sea ice concentration data from April 01 to December 31, 2019 were obtained from https://www.meereisportal.de (grant: REKLIM-2013-04)**.** The online sea-ice knowledge and data platform meereisportal.de is used with permission from Alfred Wegener Institute for Polar and Marine Research & German Society of Polar Research.

Supplementary Tables

Supplementary Table 1 Summary of data sets and references used for back trajectory modeling by surface type.

Dataset name	Description	Resolution	Source
CCI Land Cover	Copernicus Climate Change Service - Global Land Cover map	annually, 2018, 300m	[15]
MOD10C1 MYD10C1	MODIS/Terra & Aqua Snow Cover Daily L3 Global 0.05Deg CMG - Global percentage of snow-covered land and cloud-covered land	daily, February 2018 - August 2019, 0.05°	[16]
AMSR-2	Advanced Microwave Scanning Radiometer 2 - Global sea ice concentrations	daily, February 2018 - August 2019, 10km	[17]

Supplementary Table 2. **Estimate of Pan-Arctic coastal sediment erosion.** The budget is based on coastal mass and organic carbon (OC) erosion from [18] and mineral soil Hg/OC ratios from [19,20]. A formal uncertainty budget cannot be evaluated due to lack of reported uncertainties in [18], but is on the order of 50% (1σ) for the total (Tot) OC and Hg erosion budgets. BS, backshore.

	BS elevation	erosion rate	OC	ground ice	Density*	Length	Hg/OC**	Tot OC eroded	Tot Hg eroded
	m	m yr^{-1}	%	%	Mg m^{-3}	km	Mg Tg^{-1}	Tg yr^{-1}	Mg yr^{-1}
Russian Chukchi Sea	14.54	0.27	1.09	13.9	1.46	2736	0.62	0.15	0.09
USA Chukchi Sea	4.98	0.49	3.78	23.99	1.23	4662	0.62	0.40	0.25
USA Beaufort Sea	1.54	1.15	5.70	26.92	1.09	3376	0.62	0.27	0.17
Canadian Beaufort Sea	6.74	1.12	2.43	29.42	1.34	5672	0.62	0.99	0.61
CAA	No data	0.01	1.87	14.23	1.39	4656	0.62	0.00	0.00
Svalbard	13.96	0	2.86	0	1.31	8782	0.62	0.00	0.00
Barents Sea	10.52	0.42	0.92	16.22	1.48	17965	1.06	0.90	0.96
Kara Sea	14.04	0.68	1.51	23.65	1.42	25959	1.06	4.06	4.31
Laptev Sea	11.91	0.73	1.63	17.13	1.41	16927	0.70	2.81	1.97
East Siberian Sea	8.79	0.87	1.64	19.62	1.41	8942	0.70	1.27	0.89
Total									**9.25**

* Bulk density was estimated from the observed mineral soil density vs. OC% for the Canadian Arctic by [21]; their mean density derived in Table S2 of 1.36 Mg m^{-3} agrees well with Siberian glacial sediment mean density of 1.23 Mg m^{-3} by [22].

** A Hg/OC ratio of 0.62 ± 0.23 Mg Tg^{-1} is applied to N-American and Russian Chukchi Sea coasts, based on [20]; a value of 1.06 ± 0.24 Mg Tg^{-1} is applied to Barents and Kara Seas, based on mineral soil Hg/OC in the two northernmost (65.9° and 67.4° N) Siberian permafrost cores from [19]; a value of 0.70 Mg Tg^{-1} is applied to Laptev and East Siberian Seas, based on Bykovsky Peninsula deep glacial sediment cores with mean OC of 2.13 wt%, and Hg of 15 ng g^{-1} from [22].

Supplementary References

1. Demers, J. D., Blum, J. D. & Zak, D. R. Mercury isotopes in a forested ecosystem: Implications for air-surface exchange dynamics and the global mercury cycle. *Glob. Biogeochem. Cycles* **27**, 222–238 (2013).
2. Demers, J. D., Sherman, L. S., Blum, J. D., Marsik, F. J. & Dvonch, J. T. Coupling atmospheric mercury isotope ratios and meteorology to identify sources of mercury impacting a coastal urban-industrial region near Pensacola, Florida, USA. *Glob. Biogeochem. Cycles* **29**, 1689–1705 (2015).
3. Gratz, L., Keeler, G., Blum, J. & Sherman, L. S. Isotopic composition and fractionation of mercury in Great Lakes precipitation and ambient air. *Environ. Sci. Technol.* **44**, 7764–7770 (2010).
4. Enrico, M. *et al.* Atmospheric mercury transfer to peat bogs dominated by gaseous elemental mercury dry deposition. *Environ. Sci. Technol.* **50**, 2405-2412 (2016).
5. Yu, B. *et al.* Isotopic Composition of Atmospheric Mercury in China: New Evidence for Sources and Transformation Processes in Air and in Vegetation. *Environ. Sci. Technol.* **50**, 9262–9269 (2016).
6. Fu, X., Marusczak, N., Wang, X., Gheusi, F. & Sonke, J. E. Isotopic Composition of Gaseous Elemental Mercury in the Free Troposphere of the Pic du Midi Observatory, France. *Environ Sci. Technol.* **50**, 5641–5650 (2016).
7. Sherman, L. S. *et al.* Mass-independent fractionation of mercury isotopes in Arctic snow driven by sunlight. *Nat. Geosci.* **3**, 173–177 (2010).
8. Obrist, D. *et al.* Tundra uptake of atmospheric elemental mercury drives Arctic mercury pollution. *Nature* **547**, 201 (2017).
9. Campeau, A. *et al.* Sources of riverine mercury across the Mackenzie River Basin; inferences from a combined HgC isotopes and optical properties approach. *Sci. Total Environ.* **806**, 150808 (2022).
10. Jiskra, M., Sonke, J. E., Agnan, Y., Helmig, D. & Obrist, D. Insights from mercury stable isotopes on terrestrial-atmosphere exchange of Hg(0) in the Arctic tundra. *Biogeosciences* **16**, 4051–4064 (2019).
11. Zheng, W. *et al.* Mercury stable isotopes reveal the sources and transformations of atmospheric Hg in the high Arctic. *Appl. Geochem.* **131**, 105002 (2021).
12. Sonke, J. E. *et al.* Mercury stable isotope composition of lichens and mosses from northern Eurasia. Preprint at [https://eartharxiv.org/repository/view/3451/] (2022)
13. Jiskra, M. *et al.* Mercury stable isotopes constrain atmospheric sources to the ocean. *Nature* **597**, 678–682 (2021).

14. Schlitzer, Reiner, Ocean Data View, https://odv.awi.de (2021).

15. Defourny, P. *et al.* Copernicus Climate Change Service – Product User Guide and Specification ICDR Land Cover 2016-2020, Louvain. (2018).

16. Hall, D. K. & Riggs, G. A. MODIS/Terra Snow Cover Daily L3 Global 0.05Deg CMG, Version 61. Boulder, Colorado USA. NASA National Snow and Ice Data Center Distributed Active Archive Center (2021).

17. Lavelle, J., Tonboe, R. & Tian, T. Product User Manual for the OSI SAF AMSR-2 Global Sea Ice Concentration. Product OSI-408. Danish Meteorological Institute (2016).

18. Lantuit, H. *et al.* The Arctic Coastal Dynamics Database: A New Classification Scheme and Statistics on Arctic Permafrost Coastlines. *Estuaries Coasts* **35**, 383–400 (2012).

19. Lim, A. G. *et al.* A revised northern soil Hg pool, based on western Siberia permafrost peat Hg and carbon observations. *Biogeosciences* **17**, 3083–3097 (2020).

20. Olson, C. L., Jiskra, M., Sonke, J. E. & Obrist, D. Mercury in tundra vegetation of Alaska: Spatial and temporal dynamics and stable isotope patterns. *Sci. Total Environ.* **660**, 1502–1512 (2019).

21. Hossain, M. F., Chen, W. & Zhang, Y. Bulk density of mineral and organic soils in the Canada's arctic and sub-arctic. *Inf. Process. Agric.* **2**, 183–190 (2015).

22. Rutkowski, C. *et al.* Mercury in sediment core samples from deep Siberian ice-rich permafrost. *Front. Earth Sci.* **9**, 752 (2021).
